# Antiviral Activity of a Nanoemulsion of Polyprenols from Ginkgo Leaves against Influenza A H3N2 and Hepatitis B Virus *in Vitro*

**DOI:** 10.3390/molecules20035137

**Published:** 2015-03-19

**Authors:** Cheng-Zhang Wang, Wen-Jun Li, Ran Tao, Jian-Zhong Ye, Hong-Yu Zhang

**Affiliations:** 1 Institute of Chemical Industry of Forestry Products, CAF, Nanjing 210042, China; E-Mails: liwenjun611@163.com (W.-J.L.); tramoon1949@163.com (R.T.); yejianzhong1984@163.com (J.-Z.Y.); chemicalzhy@163.com (H.-Y.Z.); 2 Nation Engineering Lab. for Biomass Chemical Utilization, Nanjing 210042, China; 3 Key and Open Lab. on Forest Chemical Engineering, SFA, Nanjing 210042, China

**Keywords:** polyprenols, ginkgo leaves, nanoemulsion, influenza A H_3_N_2_ virus, hepatitis B virus, antiviral activity, *in vitro*

## Abstract

In order to improve the bioavailability levels of polyprenols (derived from ginkgo leaves (GBP)) in the human body, a GBP nanoemulsion was prepared, and its antiviral activity was evaluated against influenza A H3N2 and hepatitis B virus *in vitro*. Methods: A GBP nanoemulsion was prepared by inversed-phase emulsification (IPE). Next, we investigated the antiviral activity of the GBP nanoemulsion on influenza A H3N2 and hepatitis B virus *in vitro* by the MTT (3-(4,5-dimethylthiazol-2-yl)-2,5-diphenlytetrezolium bromide) method. ELISA and the fluorescent quantitative PCR method were used to measure the content of HBsAg, HBeAg and DNA virus in human samples. Results: The GBP nanoemulsion exhibited uniformity at an average particle size 97 nm with a hydrophilic-lipophilic balance (HLB) of 9.5. GBP is non-toxic to normal cells, hepatitis B virus DNA, hepatitis B virus antigen and HepG2215. Furthermore, GBP could reach a 70% **v**irucidal activity and a 74.9% protection rate (*** *p* < 0.001) on MDCK cells infected with H_3_N_2_ virus at a high concentration of 100 μg/mL. GBP had a good inhibition rate on HBsAg (52.11%, ** *p* < 0.01) at 50 μg/mL and Day 9 of incubation, and a 67.32% inhibition effect on HBeAg at a high concentration of 100 μg/mL and Day 9. GBP had good inhibition on HBV DNA with CT 18.6 and lower copies (** *p* < 0.01) at a middle concentration of 12.5 to 25 μg/mL. Conclusions: The GBP nanoemulsion was very stable and non-toxic and had very strong antiviral activity against influenza A H_3_N_2_ and hepatitis B virus *in vitro*. The inhibitory effects and reactive mechanisms were similar to the drug, 3TC; by lengthening the incubation time and increasing the drug concentration, GBP has promising potential as an antiviral drug.

## 1. Introduction

Influenza virus causes considerable disease epidemics due to the rapid mutation of its genes, frequent variations and surface antigens. The predominant circulating influenza A virus has almost become the influenza H3N2 subtype, because the virus is able to cause epidemic pathogens frequently and common across the globe [1]. Hemagglutinin (HA) and nerve neuraminidase (NA), which are surface markers of the influence virus, mutate on a frequent basis, and thus, no effective vaccines or drugs have been developed to treat the occurrence of this virus [2]. Hepatitis B virus (HBV) is the most common hepatitis virus in China, affecting up to 350 million persons across China. Chronic HBV infection significantly increases the risk for the development of cirrhosis, hepatic failure and hepatocellular carcinoma (HCC); in China, up to one million deaths per year can be attributed to cirrhosis and HCC [3]. Severe reactivation of hepatitis B virus can be considered a key factor leading to chronic hepatitis B, cirrhosis and HCC [4]. For persons with a high HBV DNA level, antiviral therapy should be administered. A number of nucleoside analogues have been developed as antiviral drugs. However, antibiotics, interferon and nucleoside analogues have serious side effects with long-term use for those patients that take them; it is very important to probe for new types of antiviral agents from natural products, especially those that have high efficacy on resistant mutant viral strains and low toxicity to the host.

**Figure 1:**
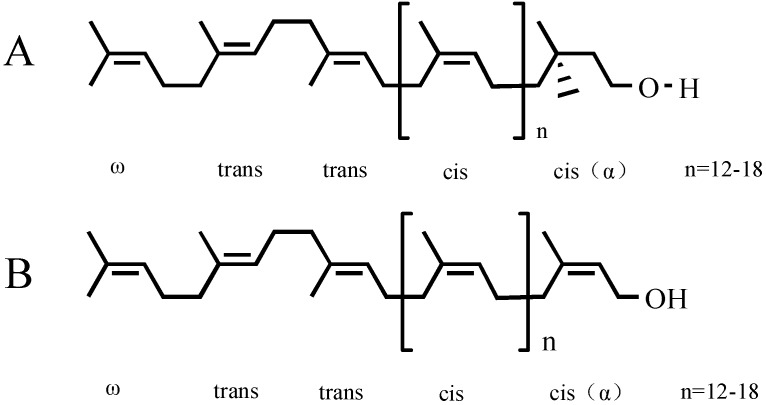
Chemical structure of dolichol and polyprenol of *Ginkgo biloba* L. (**A**) Dolichol; (**B**) polyprenols of *Ginkgo biloba* L. (GBP).

Plant polyprenols (PP) exist widely in angiosperms and gymnosperms. PP families may serve as chemotaxonomic markers for systematic families in botanic taxonomy [5]. PP and their phosphate esters can be translated into dolichol and dolichyl phosphate via enzyme catalase and form the dolichol phosphate (DHP) cycle [6], which primarily takes part in the biosynthesis of various glycoproteins that make up part of the biomembrane structure *in vivo* as a key carrier of glycoprotein [7]. A great deal of bioactive substances have been founded in *Ginkgo biloba* L., such as flavonoids, terpene lactones and polyprenols. The extract of *Ginkgo biloba* L. (EGB) and its preparations have already been widely used to treat or to prevent cardiovascular and cerebrovascular diseases in the clinical setting, due to the strong pharmacological effect of flavonoids and terpene lactones [8]. Polyprenols of *Ginkgo biloba* L. (GBP), as novel natural active lipids, were discovered after ginkgo flavonoids and terpene lactones, are generally composed of 16 to 22 unsaturated isoprene units and belong to the betulaprenol type of ω-(trans)_2_-(cis)-n-(cisα) with α-cis isoprene unit. PP has a similar structure to dolichol with the same isoprene units; however, dolichol instead has α-saturated 2,3-dihydropolyprenol (Figure 1) [9].

PP can be considered as a “label” that grants the possibility to the innate immune system to recognize infection at the early stages and govern the acquired immunity [10]. Polyprenols and polyprenyl phosphates both act as wide-spectrum antiviral agents with efficient therapeutic rates ranging from 60% to 90% [11] and greatly influence the immune system [12]. Polyprenols were developed as a prototype anti-influenza in aerosol form on the basis of *Abies sibirica* [13], and aerosol formulation of polyprenols was evaluated for the prophylactic efficacy and the mechanism of influenza infection [14,15]. At the same time, the injection formulation of polyprenols was also developed as an immunostimulator to decrease the level of macrophages and to significantly increase lymphocytes; the polyprenol emulsions had a relatively low hydrophilic-lipophilic balance and could inhibit influenza virus infection in mice through a modulation of the host immune response [16]. A bioactive drug, ropren, had been developed from pine needle polyprenols in Russia and Austria, and the effect of ropren had been studied on the key enzymes of the cholinergic and monoaminergic types of nervous transmission [17]. In our earlier studies, GBP showed antibacterial activity against *Salmonella enterica*, *Staphylococcus aureus*, *Aspergillus niger*, *Escherichia coli* and *Bacillus subtilis* [18,19]. Wang *et al.* found that GBP had a powerful protective effect on acute hepatic injury induced by carbon tetrachloride and alcohol [20,21,22], as well as antitumor efficacy [23,24,25]. However, there is very little in the medical literature that reports on the antiviral activity of polyprenols from ginkgo leaves against influenza A H_3_N_2_ and hepatitis B virus and, in particular, the nanoemulsion form of polyprenols from ginkgo.

Due to strong hydrophobic isoprene units in its structure, GBP is very difficult to disperse in water, which leads to a lower bioavailability in the body. Nanoemulsion has good prospects in the field of submicron emulsions due to the small particle size and long-term dynamic stability without obvious stratification or coalescence. Therefore, we propose to develop a GBP nanoemulsion to improve GBP’s hydrophilic and cell member penetrative features. In traditional medicine, there is a very long history for the use of Chinese herbs as antiviral medicine, to improve the body’s immune function with less side effects. In order to speculate about GBP’s antiviral activity and to raise its bioavailability, we prepared a GBP nanoemulsion, then tested the bioactivities of the GBP nanoemulsion on the cells by growth inhibition, killing virus directly and protecting the cells by MTT with respect to MDCK cells *in vitro*. In addition, ELISA and the fluorescent quantitative PCR method were used to measure the content of HBsAg, HBeAg and viral DNA in the supernatant. This investigation will provide the basis for the comprehensive utilization of bioactive compounds of flavonoids, terpene lactones and polyprenols from *Ginkgo biloba* leaves.

## 2. Results and Discussion

### 2.1. Determination of GBP

The yield of purified GBP was 0.35% of dried ginkgo leaves. An ethyl acetate/petroleum ether (1/9, v/v) mixture as the developing solvent was used for TLC, and the spots were detected by iodine for color development. The R_f_ value of GBP was 0.35. HPLC was performed at the wavelength detected at 210 nm with a 5-μm Kromasil C18 ODS-1 (150 mm × 4.6 mm) column, using 1.0 mL/min of an isopropanol/methanol/hexane/water mixture (50/25/10/2, v/v) as the eluent. The curve of the standard polyprenols from *Ginkgo biloba* leaves (C_75_–C_105_), which had a better linear relation between the injection volume (4.6–23.3 μg) and peak area, was expressed as the regression Equation (1):

*y* = 1.5751*x* + 1.1546 (*R*^2^ = 0.9991) (1)

**Figure 2:**
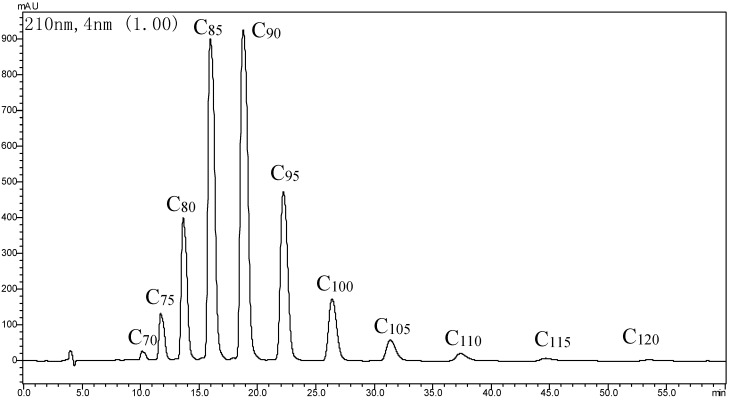
HPLC of GBP.

The retention time of GBP with different isoprene units is 10.127 min (C_70_), 11.724 min (C_75_), 13.651 min (C_80_), 15.966 min (C_85_), 18.782 min (C_90_), 22.216 min (C_95_), 26.393 min (C_100_), 31.361 min (C_105_), 37.368 min (C_110_), 44.663 min (C_115_) and 53.519 min (C_120_). The purity of GBP was 91.3%, calculated according to the curve regression equation of the standard polyprenols, whereas, C_85_ and C_90_ polyprenols were dominant (Figure 2).

### 2.2. Physical Properties of GBP Nanoemulsion

The stability of the GBP emulsion was affected by the key factors of the hydrophilic-lipophilic balance (HLB) value and particle size. The physical properties of the GBP emulsion could be evaluated by centrifugation stability, dispersity, storage stability and the number of refrigeration cycles. When the GBP emulsion had an HLB value of 8.5 to 11.5, it produced a relatively stable nanoemulsion with average diameters between 389 nm to 988 nm. In particular, when the HLB value was between 9.0 to 10, the GBP nanoemulsion did not cause stratification after centrifugation; the dispersity and storage stability both reached the one-level; centrifugation stability approached the two-level; the refrigeration cycle could be repeated three times; and it showed good uniformity. When the HLB value was less than 8.5 or over 11, the GBP emulsion emerged as uneven in density with two levels of dispersity and storage stability.

**Figure 3:**
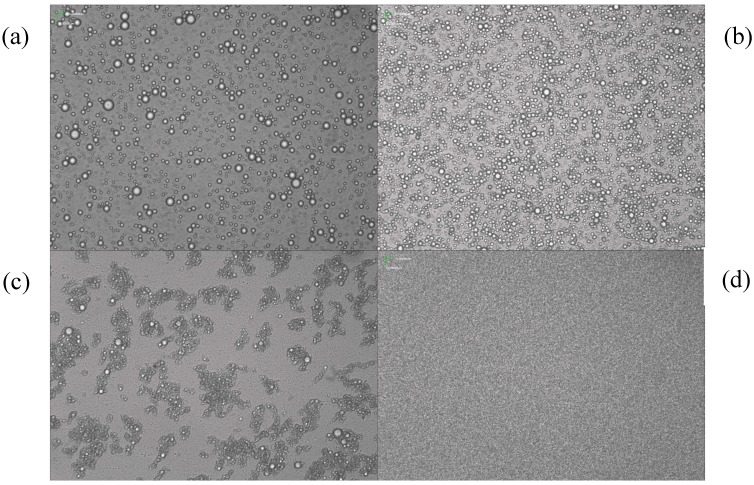
The microscopic image of the GBP nanoemulsion under different conditions: (**a**) 15 min, 75 °C, 20,000 r/min; (**b**) 10 min, 60 °C, 10,000 r/min; (**c**) 5 min, 45 °C, 10,000 r/min; (**d**) 12 min, 60 °C, 17,000 r/min (a, b, c, d ×400).

**Figure 4:**
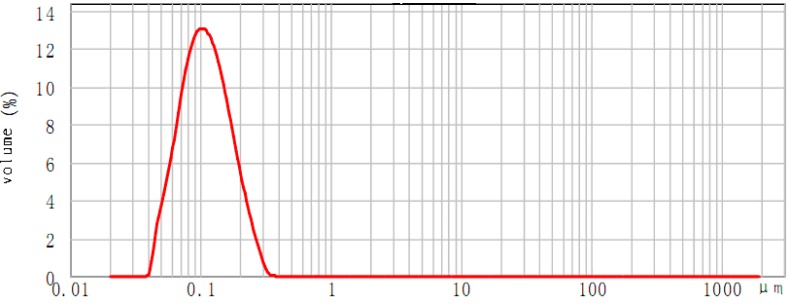
The particle diameter distribution of the GBP nanoemulsion under the optimum conditions.

The GBP emulsion particle size and its stability changed with different emulsification parameters, *i.e*., stirring speed, the emulsifying temperature and time and the ratio of emulsifiers to GBP (mL/g). The microscopic morphology of the GBP nanoemulsion was investigated under different single factors. The GBP emulsion showed a definite uneven distribution of particle sizes between 389 nm to 988 nm, shown in Figure 3a–c, while Figure 3d exhibits very good uniformity. Therefore, based on the single factor and response surface method (RSM) of Figure 3d, when the HLB value was 9.5, the GBP nanoemulsion was optimized for a mixing time of 12 min, an emulsifying temperature of 60 °C and a stirring speed 17,000 r/min; then, the GBP nanoemulsion reached an average particle size of 97 nm (Figure 4).

### 2.3. Cellular Toxic Effect of GBP

When MDCK and HepG 2215 cells were exposed to different concentration of GBP ranging from 0.01 to 100 μg/mL for 48 h separately, the shape of the cells was observed every day by microscope. No abnormal cell morphology nor cell disruption were discovered. Compared to normal controls, the survival rate of all GBP groups was over 100% (toxic concentration (TC) >1, IC = 0), and the cell breakage rates of the GBP groups were all negative. GBP did not have a significant influence on the shape and disruption of the cells, and GBP had no cytotoxic effect on the proliferation of MDCK and HepG 2215 cells at the concentration range of 0.01 to 100 µg/mL. As shown in Table 1, GBP did not inhibit cell growth.

**Table 1:** Cell toxicity of GBP on MDCK and HepG 2215 cells (χ ± s, n = 4). TC, toxic concentration.

GBP Concentration	MDCK Cell			HepG 2215 Cell			IC	*p*
	OD_570_	cell Breakage Rate	TC	OD_570_	Cell Breakage Rate	TC		
100 μg/mL	3.214 ± 0.300	−12.93	1.129	1.873 ± 0.300	−1.849	1.018	0	>0.05
10 μg/mL	3.571 ± 0.067	−25.48	1.255	2.111 ± 0.067	−14.80	1.148	0	>0.05
1 μg/mL	2.869 ± 0.294	−0.808	1.008	1.970 ± 0.294	−7.127	1.071	0	>0.05
0.1 μg/mL	3.236 ± 0.164	−13.71	1.137	2.139 ± 0.164	−16.32	1.163	0	>0.05
0.01 μg/mL	3.675 ± 0.153	−29.14	1.291	1.807 ± 0.153	1.741	0.983	0	>0.05
Control 0 μg/mL	2.846 ± 0.121			1.839 ± 0.121			/	/

### 2.4. Effect of GBP on Influenza Virus and Normal Cells

To investigate the affection of GBP on influenza virus and normal cells, the TCID_50_ of H_3_N_2_ influenza virus was evaluated after treatment for 1 h with various concentrations of GBP and ribavirin; the results are shown in Table 2. The result indicated that cells positive for ribavirin had very strong virucidal activity with a 97.4% inhibition rate (*** *p* < 0.001) on the TCID_50_ of H_3_N_2_ influenza virus and provided a protective effect for MDCK normal cells with a protection rate of 92.3% (*** *p* < 0.001). The median effective concentrations (IC_50_) of GBP were 3.179 and 1.408, respectively, on influenza virus and normal cells, which displayed that GBP had a better protecting effect than virucidal activity with the increase of the GBP concentration. When the GBP concentration exceeded 25 μg/mL, the statistical difference is very obvious (** *p* < 0.01) at the middle concentration of 50 μg/mL. GBP reached 65.9% virucidal activity against the H3N2 influenza virus and a 60.4% protection rate for MDCK cells, particularly at the high concentration of 100 µg/mL. GBP was able to reach 70% virucidal activity and a 74.9% protection rate. Therefore, the experiment resulted in a suggested increased concentration of GBP that could raise the efficacy of GBP on influenza virus and normal cells.

**Table 2:** Effect of GBP on H3N2 influenza virus and MDCK cells *(*χ ± s, n = 4).

Groups	Cons. (μg/mL)	Virucidal Activity			Protecting MDCK Cells		
		OD_570_	Inhibition Rate (%)	IC_50_	OD_570_	Protection Rate (%)	IC_50_
GBP+ H3N2 virus	100	1.729 ± 0.238 **	70.0		1.683 ± 0.201 **	74.9	
GBP+ H3N2 virus	50	1.691 ± 0.192 **	65.9		1.454 ± 0.142 **	60.4	
GBP+ H3N2 virus	25	1.391 ± 0.271 **	49.5	3.179	1.422 ± 0.302 **	58.2	1.408
GBP+ H3N2 virus	12.5	0.861 ± 0.318	20.5		1.103 ± 0.398 *	37.9	
GBP+ H3N2 virus	6.2	0.507 ± 0.089	1.1		0.632 ± 0.223	8.0	
Ribavirin+ H3N2 virus	10	2.267 ± 0.193 ***	97.4		1.957 ± 0.282 ***	92.3	
MDCK *cell control*	–	2.315 ± 0.213			2.078 ± 0.104		
H3N2 virus control	–	0.486 ± 0.098			0.507 ± 0.205		

Note: * *p* < 0.05, ** *p* < 0.01, *** *p* < 0.001, compared to the virus control.

### 2.5. Inhibition Effect of GBP on HBV Antigen and DNA

At 3 d, 6 d and 9 d of incubation treatment with different concentrations of GBP, the inhibitory effect on HBsAg and HBeAg secreted by HepG 2215 cells in the culture medium was measured. In Table 3, GBP had a better dose-effect inhibitory relation and time-effect inhibitory relation on HBsAg, as a general trend. The inhibition effect could be increased by elongation of the incubation time and increasing the GBP concentration. The positive control drug (3TC, 20 µg/mL) had a very strong inhibition rate on HBsAg (61.99%) and HBeAg (69.99%) (** *p* < 0.01). After a 9-d incubation period, GBP could reach a 41.40% and 52.11% inhibition rate on HBsAg (** *p* < 0.01) at a high concentration of 100 µg/mL, respectively at Day 3 and Day 6, while the IC_50_ of GBP was 42.71, 7.450 and 5.531 µg/mL, respectively, at Day 3, Day 6 and Day 9. Therefore, the inhibition effect of GBP was best on HBsAg (52.11%, ** *p* < 0.01) at 50 μg/mL and Day 9; however, it was still distinctly weaker than positive 3TC in Table 3. GBP had a better obvious inhibition effect on HBeAg than on HBsAg at the high concentration of 100 µg/mL, and reached 48.45%, 48.85% and 67.32%, respectively, at Days 3, 6 and 9; in particular, on Day 9, the IC_50_ of GBP arrived at the minimum value of 5.349 µg/mL, in which GBP had a similar inhibition effect as positive 3TC, while at low concentration of GBP and 3 d of incubation, there was a very weak effect (*p* > 0.05) in Table 4.

In fact, the best inhibition against HBsAg secretion was found to be on Day 6 at a high dose of GBP (100 µg/mL) and on Day 9 at middle dose of GBP (100 µg/mL), which implies that a high concentration of GBP could reverse the inhibitory effect on HBsAg secretion. This discrepancy might be caused by the structure of GBP and its nanoemulsion feature. GBP has high molecular weights and strong hydrophobic isoprene units, which resulted in a lower bioavailability in the body. Meanwhile, a high concentration of GBP restricted GBP’s dispersion in the emulsion; therefore, GBP was not able to penetrate the cell membrane. Further investigation is required in the future.

**Table 3:** Inhibitory effect of GBP on HBsAg secreted by HepG 2215 cells (χ ± s, n = 4).

Groups	OD_450_			Inhibition Rate (IC), %			IC_50,_ μg/mL		
	Day 3	Day 6	Day 9		Day 3	Day 6	Day 9		Day 3
GBP 100 μg/mL	0.542 ± 0.076	0.794 ± 0.510 *	0.814 ± 0.132	41.405	52.110	38.380			
GBP 50 μg/mL	0.678 ± 0.080	0.875 ± 0.110 *	0.634 ± 0.132	15.676	47.225	52.116			
GBP 25 μg/mL	0.696 ± 0.030	1.057 ± 0.120 *	0.716 ± 0.167	24.756	36.248	45.798	42.71	7.450	5.531
GBP 12 μg/mL	0.801 ± 0.108	1.311 ± 0.238	0.949 ± 0.194	13.405	20.928	28.160			
GBP 6.2 μg/mL	0.972 ± 0.195	1.416 ± 0.319	1.074 ± 0.444	5.081	14.596	18.697			
3TC 20 μg/mL	0.527 ± 0.09 **	0.663 ± 0.137 **	0.502 ± 0.193 **	43.027	60.012	61.998			
cell control	0.925 ± 0.062	1.658 ± 0.065	1.321 ± 0.073	–	–	–			

Note: * *p* < 0.05, ** *p* < 0.01, compared to the control.

**Table 4:** Inhibitory effect of GBP on HBsAg secreted by HepG 2215 cells (χ ± s, n = 4).

Groups	OD_450_			Inhibition Rate (IC), %			IC_50,_ μg/mL		
	Day 3	Day 6	Day 9		Day 3	Day 6	Day 9		Day 3
GBP 100 μg/mL	0.225 ± 0.038	0.222 ± 0.194	0.493 ± 0.166 *	48.456	48.848	67.32			
GBP 50 μg/mL	0.356 ± 0.049	0.346 ± 0.073	0.824 ± 0.131 *	17.972	20.276	45.394			
GBP 25 μg/mL	0.392 ± 0.029	0.382 ± 0.158	1.068 ± 0.179 *	9.677	11.981	29.225	59.02	46.55	5.349
GBP 12 μg/mL	0.402 ± 0.044	0.392 ± 0.056	1.159 ± 0.290	7.373	9.677	23.194			
GBP 6.2 μg/mL	0.426 ± 0.037	0.416 ± 0.046	1.265 ± 0.187 *	1.843	4.147	16.170			
3TC 20 μg/mL	0.31 ± 0.035 *	0.310 ± 0.057 *	0.483 ± 0.134 **	28.571	28.571	69.992			
cell control	0.434 ± 0.040	0.434 ± 0.113	1.509 ± 0.105	–	–	–			

Note: * *p* < 0.05, ** *p* < 0.01, compared to the control.

**Figure 5:**
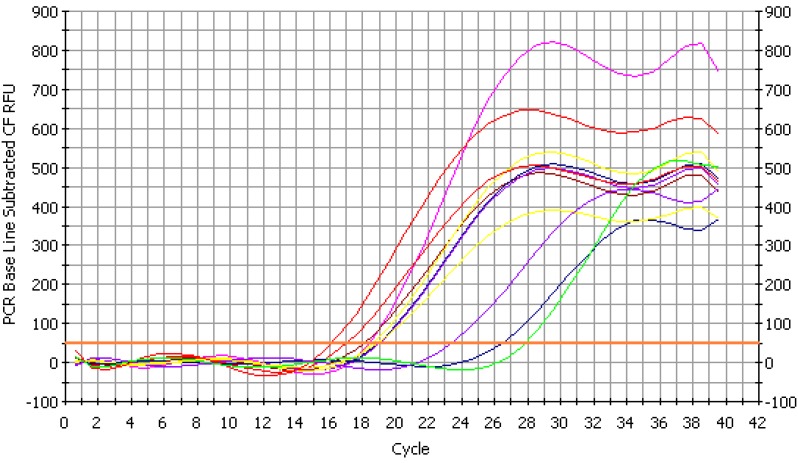
Fluorescence quantitative curve of ICycler.

A quantitative assay was performed on the extracted HBV DNA using quantitative fluorescence PCR (FQ-PCR) using the sequence numbers 5'-AAC TGA AAG CCA AAC AGT G-3' and 5'-CCT CTT CAT GCT GCT-3'. The fluorescence quantitative curve of ICycler is shown in Figure 5; the quantitative DNA of HBV is shown in Table 5. The results indicated that the drug, 3TC, had a very strong inhibitory effect on HBV DNA with CT 22.9 and copies of 5.04 × 10^−3^ (** *p* < 0.01), and GBP showed good inhibition of HBV DNA, particularly in the middle concentrations of 12.5 μg/mL to 25 μg/mL, with CT 18.6 and lower copies (** *p* < 0.01).

**Table 5:** Quantitative DNA of HBV.

Concentrations of Drug	Groups	CT	Copies/μL
GBP 100 μg/mL	F1	18.6	5.32 × 10^4^ **
GBP 50 μg/mL	F2	18.6	5.49 × 10^4^ **
GBP 25 μg/mL	F3	18.2	7.08 × 10^4^ *
GBP 12.5 μg/mL	F4	18.1	7.60 × 10^4^ *
GBP 6.2 μg/mL	F5	17.7	1.46 × 10^4^ *
3TC 20 μg/mL	F6	22.9	5.04 × 10^4^ **
No Treatment	F7	16.4	2.28 × 10^4^

Note: * *p* < 0.05, ** *p* < 0.01, compared to the control.

## 3. Experimental Section

### 3.1. Chemicals and Instruments

Standard polyprenols from *Ginkgo biloba* leaves (C_75_–C_105_) were purchased from Larodan Fine Chemical Co., Ltd, (Shanghai, China), Sweden. Ninety six-well cell culture plates were obtained from Corning. DMEM culture medium was bought form Gibco-BRL (New York, NY, USA). FBS was provided by Hyclone. Diagnostic kits for HBsAg (colloidal gold) and HBeAg (colloidal gold) were both provided by Shanghai Kehua Bio-engineering Co., Ltd (KHB). (Shanghai, China) Isolation kits for viral DNA were bought from Shanghai Zhongke Technology Co., Ltd. (Shanghai, China) Taq DNA polymerase, d-NTP and the fluorescence quantitative PCR detection kit were provided by Takara. MTT (3-(4,5-dimethylthiazol-2-yl)-2,5-diphenyl tetrazolium bromide) was from Sigma Co. (Shanghai, China) The Benchmark Plus ELISA, and the ICycler fluorescent quantitative PCR were both supplied by Bio-Rad (Hercules, CA, USA).

### 3.2. Viruses, Cells and Positive Control Compounds

The influenza A H3N2 virus strain and HepG 2215 cells were obtained from Fudan University (Yueda Biotechnology Co., Ltd.). (Shanghai, China) These cultures were derived from HepG2 cells and produced elevated levels of HBeAg and HBsAg. MDCK and HepG 2215 cells were cultured in complete DMEM (containing 10% FBS, 100 kU/L benzylpenicillin, streptomycin, G-418, L-glutamine 0.03%, pH 7.0) in 75-cm^2^ tissue culture flasks at 37 °C in a humidified 5% CO_2_. Ribavirin and lamivudine (3TC), two medicines used clinically for treating viral infections, were bought from GlaxoSmithKline (GSK) as positive control compounds.

### 3.3. Nanoemulsion and HPLC of GBP

A 1-kg amount of powdered *Ginkgo biloba* leaves underwent extraction with petroleum ether at room temperature for 48 h to get the crude lipid extract. Then, the extract was saponified at 70 °C for 1 h, with 95% ethanol containing 15% (w/v) of NaOH and 0.5% of pyrogallol. The unsaponifiable matter was extracted 4 times with petroleum ether. The organic phases collected were evaporated and the residue dissolved in a mixture (acetone/methanol = 85:15, v/v), then refrigerated for 4 h at −10 °C; the filtrate was further purified to give GBP by flash column chromatograph (200 mesh silica gel, *Φ* 2.5 × 40 cm), using petroleum ether (400 mL) and 5% ethyl ether/petroleum ether (v/v, 300 mL), as the eluent. Purified GBP (10 g) was dissolved in hexane and was stabilized with emulsifier (Tween-80, Span-80, non-ionic SAA, *etc*., in ethanol) with values of hydrophilic-lipophilic balance (HLB) by inversed phase emulsification (IPE) at 60 °C and 17,000 r/min with a mixing time of 12 min. The GBP nanoemulsion reached an average particle size 97 nm with HLB 9.5; the emulsion could be kept at 4 °C and diluted to the required concentration before it was used.

HPLC was performed at room temperature on a Shimadzu SPD-10A instrument equipped with a UV detector (210 nm) and a 5-μm Kromasil C18 ODS-1 (150 × 4.6 mm) column, using 1.0 mL/min of an isopropanol/methanol/hexane/water mixture (50/25/10/2, v/v) as the eluent.

### 3.4. Cellular Toxicity Test

MDCK and HepG 2215 cells were first seeded into 96-well plates at a density of 1.0 × 10^4^ cells per mL and cultured in 200 μL complete DMEM containing 10% FBS. After 24 h of incubation, the cells were washed with phosphate buffered saline (pH 7.0) and treated with the GBP nanoemulsion at various concentrations of GBP (100 μg/mL, 10 μg/mL, 1 μg/mL, 0.1 μg/mL, 0.01 μg/mL) for 2 d. MTT solution (5 mg/mL in phosphate buffered saline (PBS)) was added (10 μL/ well) and incubated at 37 °C for an additional 4 h. The supernatant was carefully removed, then 150 μL of DMSO were added to each well and shaken in order to dissolve the added DMSO. The cytotoxicity of the GBP nanoemulsion was tested by an MTT assay as previously described [26,27]. Then, the OD absorbance of the solution was read at 570 nm. Cell breakage rates were calculated by the following formula: cell breakage rate (BR) % = 1 − (the value of the GBP group − the value of the blank group)/(the value of the negative control group − the value of the blank group) × 100%. Toxic concentration (TC) was calculated by the following formula: TC = (the value of the GBP group − the value of the blank group)/(the value of the negative control group − the value of the blank group) × 100%.

### 3.5. Virucidal Activity and Protecting Cells Tests

MDCK cells were digested by trypsin to prepare a single cell suspension. Then the concentration of cells was adjusted to 2 × 10^4^ cells/mL after counting, and they were added to a 96-well culture plate (100 μL/well) and incubated for 24 h at 37 °C with a humidified 5% CO_2_ atmosphere. Influenza A H3N2 virus of 100 TCID_50_ was treated with GBP in different concentration (100, 10, 1, 0.1, 0.01, 20 μg/mL) and 10 μg/mL positive control drug (ribavirin), respectively, and incubated for 1 hour at 37 °C. Then, the supernatants were aspirated further and incubated for 72 h after adding the influenza virus; the virucidal activity of and protection of cells by GBP were determined by the MTT assay [26]. The OD absorbance of the solution was then read at 570 nm, and the cell inhibition rates were calculated by the formula: cell inhibition rate (IR) % = 1 − (the value of the GBP group − the value of the blank group)/(the value of the negative control group − the value of the blank group) × 100%. The 50% inhibition concentration (IC_50_) is the drug concentration that is required for 50% inhibition. IC_50_ values were estimated graphically from the plots [27,28].

### 3.6. Hepatitis B Virus Antigen Inhibition Test

HepG2215 cells were digested by trypsin to prepare a single cell suspension. Then, the concentration of cells was adjusted to 2 × 0^4^ cells/mL after adding to the 24-well culture plate (1 mL/well) and incubating for 24 h at 37 °C with a humidified 5% CO_2_ atmosphere. Then, the supernatant was added to DMEM nutrient medium containing 5% FBS and co-cultured at 37 °C with a humidified 5% CO_2_ atmosphere at different concentrations of GBP (100 μg/mL, 10 μg/mL, 1 μg/mL, 0.1 μg/mL, 0.01 μg/mL, 20 μg/mL) and 3TC (positive control). The cell medium was refreshed on Days 3, 6, and 9 post-infection; the supernatants were collected with a centrifugal tube of 1.5 mL to store frozen until use. Compared with the control group, the viral antigens HBsAg and HBeAg in the supernatant were evaluated using specific ELISA kits, and the inhibition rate (OD_450_) was calculated at 450 nm by ELISA. Viral genomic DNA was extracted from the supernatants and stored at −20 °C for later use. The cell culture supernatant was quantified by fluorescence quantitative PCR [27,28,29]. Secretion inhibition rates of HBsAg and HBeAg were calculated by the following formula: inhibition rate % = (the value of the negative control − the value of the GBP group/(the negative control value − the blank control value) × 100%. The 50% inhibition concentration (IC_50_) is the drug concentration that is required for 50% inhibition.

### 3.7. Statistical Analysis

Data were expressed as the mean X ± SD by the *t*-test and statistically analyzed by one-way ANOVA using SPSS10.0 statistical software. A *p* < 0.05 was considered to be statistically significant.

## 4. Conclusions

At present, a large number of synthetic antiviral drugs, particularly antibiotics and interferon drugs, has been applied in the clinical setting, which has caused serious problems, such as drug resistance and viral variability, and these further raise concerns about the appropriate use of adamantanamines, amantadine, ribavirin and lamivudine.

Polyprenols are natural bioactive lipids that can be metabolized into dolichol phosphate *in vivo* by enzyme CTP, as an integral part of all living cells, including prokaryotic and eukaryotic cells. Polyprenols take part in the biosynthesis of glycoproteins. Polyprenols are non-toxic and very safe, in particular in their actions as wide-spectrum antiviral agents. The animal experiments showed that plant polyprenols had good inhibition and a therapeutic effect of 60% to approximately 90% on canine enteritis, canine infectious hepatitis, murine hepatitis, cat infectious gastroenteritis, cat infectious enteritis and peritonitis, bovine leukemia, rabies and distemper virus [11]. In our experiments, we focused on the prophylactic activity of GBP against hepatitis B virus and influenza virus *in vitro*. Tests results showed that GBP is non-toxic to normal cells. Furthermore, GBP showed a better protective effect on MDCK cells infected with H3N2 virus; in particular, at the high concentration of 100 μg/mL. GBP was able to reach 70% virucidal activity and a 74.9% protection rate (*** *p* < 0.001). GPB had a similar inhibition effect and reactive mechanism on the HBV antigen and DNA as positive drug 3TC, when lengthening the incubation time and increasing the GBP concentration. GBP had a good inhibition rate on HBsAg (52.11%, ** *p* < 0.01) at 50 μg/mL and Day 9 of incubation, and HBeAg (67.32%, ** *p* < 0.01) at 100 μg/mL and Day 9, as well as CT 18.6 and lower copies (** *p* < 0.01) on HBV DNA between 12.5 to 25 μg/mL. Therefore, GBP is very promising as a potential antiviral drug.

The influence of the polyprenol structure on cell membranes has been considered the key factor [30,31,32,33]. The mechanism against viral infections of related polyprenols suggests that polyprenols play an instructive role in innate immunity in the acquired immune response [12]. The physicochemical property of the polyprenol preparation demonstrates the great influence of its antiviral biological activities. GBP has high molecular weights and strong hydrophobic isoprene units, which result in a lower bioavailability in the body. The nanoemulsions of GBP can play important roles to improve the mobility, stability and permeability of the cell membrane, to reinforce membrane fusion and to regulate the structure and function of biological membranes. We supposed that GBP expressed a direct antiviral effect, while having immunomodulatory activity; the key antiviral activity of GBP was to stimulate cells to produce interferon. An additional feature of GBP is that it has a direct killing effect on the outer membrane of the virus and protects normal cells. Further research is needed to evaluate the influence of GBP on the immune system.
